# Supplementary material for: Surgery for Adult Patients with Congenital Heart Disease: Results from the European Database
Source: J Clin Med. 2020 Aug 3;9(8):2493. doi: 10.3390/jcm9082493 (PMC7464431; doi:10.3390/jcm9082493)
Supplement: Supplementary file 1 [file jcm-09-02493-s001.pdf]

Supplementary material

Figure S1. Overall surgical workload (absolute frequencies) and hospital mortality (%)

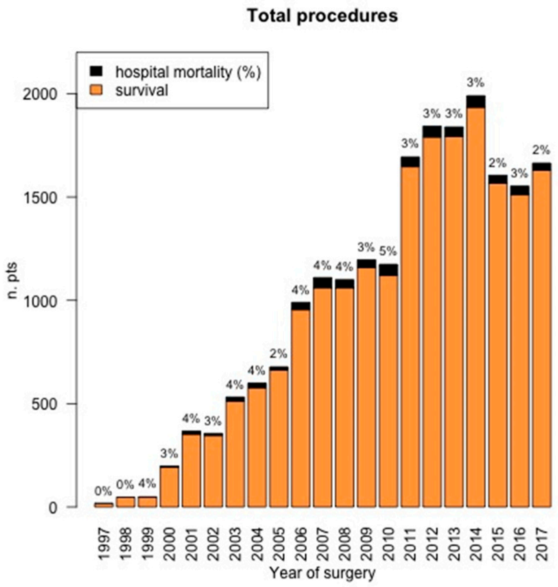

Figure S2. Surgical workload (absolute frequencies) and hospital mortality (%) according to the year of surgery for septal procedural subgroup

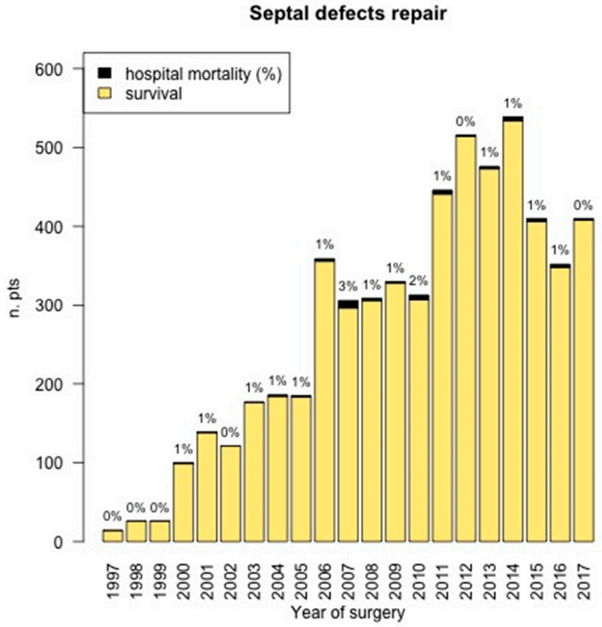

Figure S3. Surgical workload and hospital mortality (absolute frequencies) according to the year of surgery for right heart lesion procedural subgroup

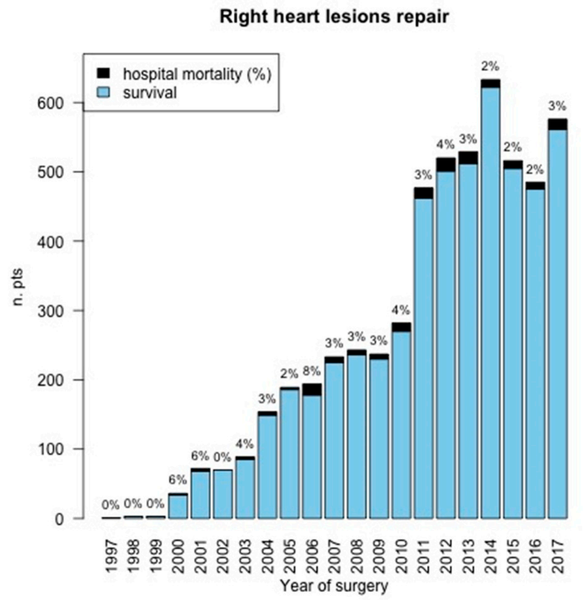

Figure S4. Surgical workload and hospital mortality (absolute frequencies) according to the year of surgery for left heart lesion procedural subgroup

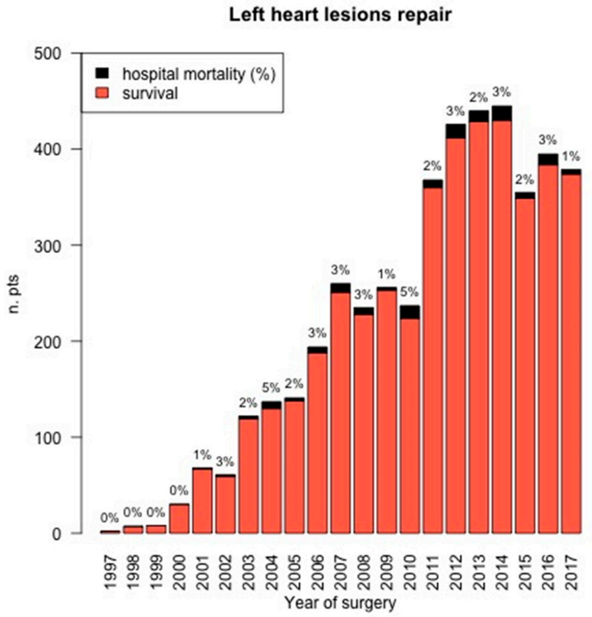

Figure S5. Surgical workload and hospital mortality (absolute frequencies) according to the year of surgery for thoracic arteries and veins procedural subgroup

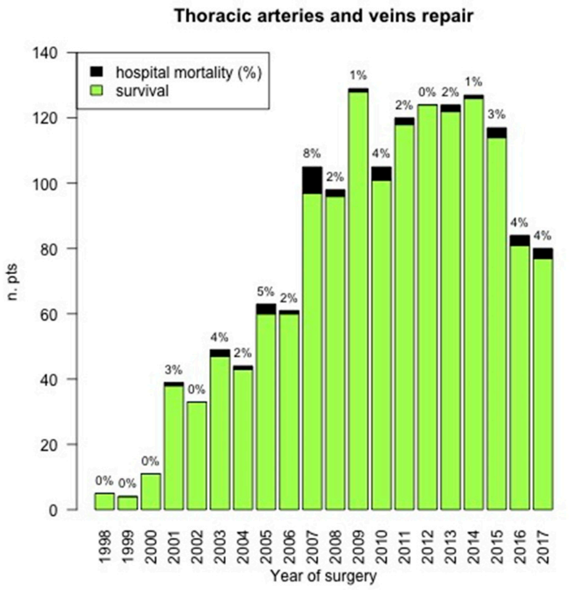

Figure S6. Surgical workload and hospital mortality (absolute frequencies) according to the year of surgery for electrophysiological procedural subgroup

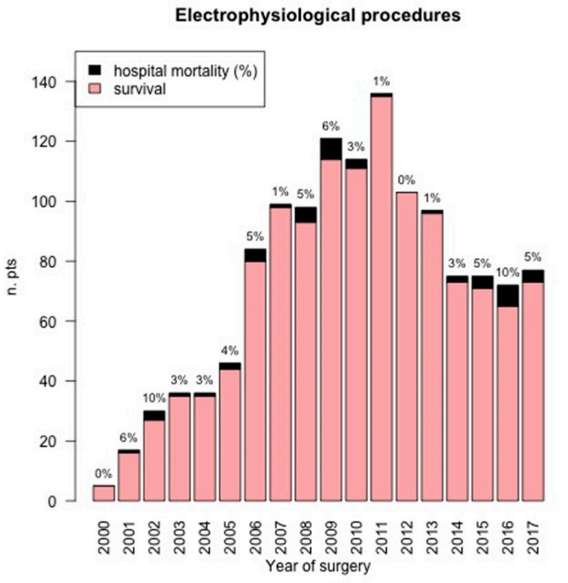

Figure S7. Surgical workload and hospital mortality (absolute frequencies) according to the year of surgery for partial anomalous pulmonary vein procedural subgroup

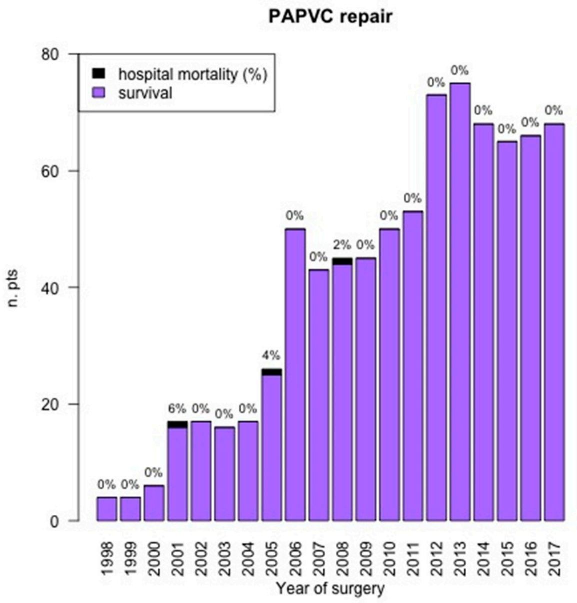

Figure S8. Surgical workload and hospital mortality (absolute frequencies) according to the year of surgery for single-ventricle associated procedural subgroup

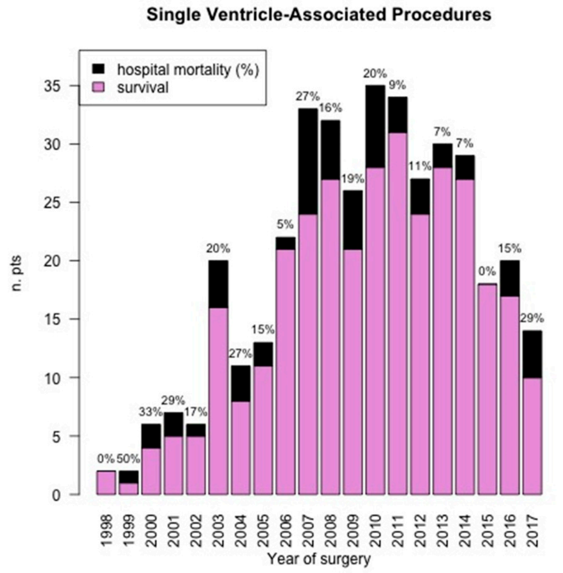

Figure S9. Surgical workload and hospital mortality (absolute frequencies) according to the year of surgery for mechanical support procedural subgroup

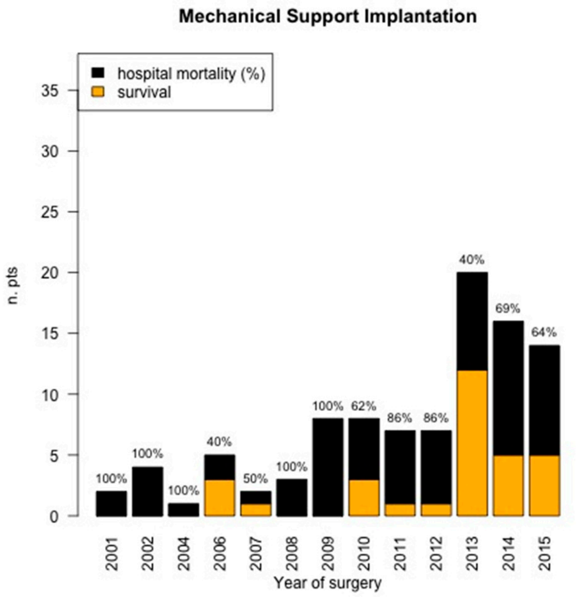

Figure S10. Surgical workload and hospital mortality (absolute frequencies) according to the year of surgery for transplants subgroup

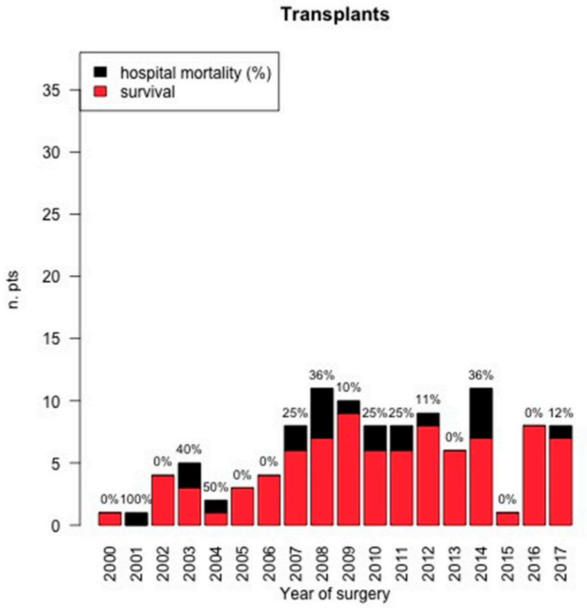

Figure S11. Surgical workload and hospital mortality (absolute frequencies) according to the year of surgery for other less common procedural subgroup

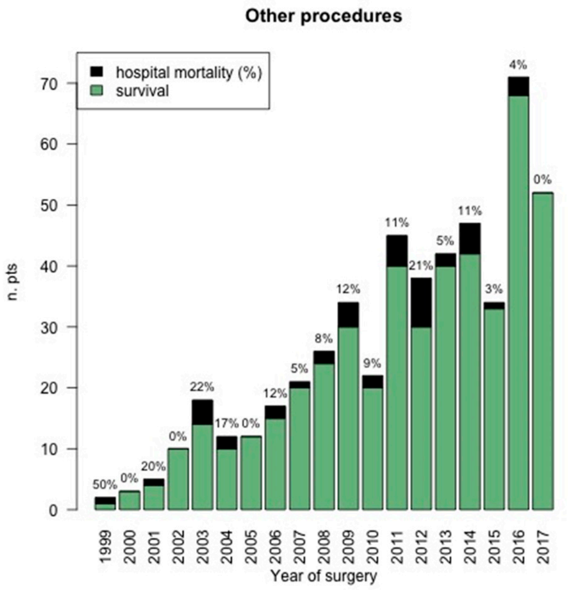

Table S1. Variable analyzed (minimal dataset from the ECHSA database)

Patient's ID
Sex (Male, Female)
Date of birth (dd/mm/yyyy)
Primary diagnosis leading to surgery
Date of surgery (dd/mm/yyyy)
Weight at the time of operation (kg)
Number of previous operations (n)
Primary operative procedure
Associated procedures
Operation type (CPB yes/no)
CPB time (min)
Aortic X-clamp time (min)
Circulatory arrest time (min)
Date of discharge from the ICU post-operation (dd/mm/yyyy)
Date of discharge/mortality (dd/mm/yyyy)
30-days status (dead, alive)

ECHSA: European Congenital Heart Surgeons Association, CPB: cardiopulmonary bypass, ICU: intensive care unit

Table S2. Isolated and associated procedures.

		Isolated procedures		Associated procedures	
Procedure	Pts	Hospital mortality	Operative risk (%)	Hospital mortality	Operative risk (%)
**Total**	**19648**	**302**	**2.6**	**289**	**3.5**
**1-Septal defects repair**	**5668**	**36**	**0.8**	**22**	**1.8**
ASD	3721	30	0.9	3	1.0
VSD	1326	5	0.8	9	0.1
AV canal (partial/intermediate)	590	1.0	0.2	7	4
ASD creation	31	-	-	3	1.0
**2- Right heart lesions repair**	**5161**	**39**	**2**	**109**	**3.2**
Tricuspid valve disease	2494	20	6.4	60	2.8
- TV plasty	2034	2	2	31	1.6
- TV replacement	297	14	9.9	23	15
- Ebstein's repair	163	4	5.5	6	6.7
Conduit operations (RV/LV to PA operation, reop, other)	1114	7	1.2	25	4.7
Pulmonary valve disease:	718	6	1.1	2	1.3
- PV replacement	661	5	0.9	1	0.9
- PV plasty	57	1	5.9	1	2.5
RVOT procedure:	502	1	0.8	14	3.7
- PA reconstruction	220	-	-	6	3.2
- 1 ½ ventricular repair	48	-	-	4	10
- DCRV repair	63	-	-	-	-
- RVOT procedure	171	1	1.8	4	3.5
TOF repair	333	5	3.1	8	4.6
**3-Left heart lesions repair**	**4280**	**50**	**2.0**	**65**	**3.7**
Aortic valve disease:	3180	30	1.4	30	2.9
- AoV replacement	1687	11	1.0	17	2.7
- Aortic root replacement	748	13	3.0	9	3.8

- Aortic stenosis sub-/supra-valvar	304	3	1.1	1	2.4
- Ross/Konno/Ross-Konno procedure	213	1.0	0.6	2	3.4
- AoV plasty	228	1.0	0.7	1	1.3
Mitral valve disease:	1100	20	5.1	35	4.9
- MV plasty	574	5	2.5	3	0.8
- MV replacement	521	15	8.0	32	9.6
- Supravalvular mitral ring	5	-	-	-	-
**4-Thoracic arteries and veins anomalies repair**	**1474**	**10**	**1.6**	**25**	**2.9**
Aortic aneurysm repair	611	1.0	1.0	11	2.2
Coarctation of aorta	348	3	1.0	1.0	1.6
CABG	259	-	-	9	3.9
Sinus of Valsalva aneurysm	59	-	-	-	-
Vascular ring repair	56	2	4	1.0	17
Patent ductus arteriosus closure	44	-	-	/	/
Anomalous origin of coronary artery from pulmonary artery repair	37	1.0	3.8	-	-
Aortic arch repair	31	2	6.7	/	/
Aortic dissection repair	27	1.0	13	3	16
Anomalous aortic origin of coronary artery repair	2	/	/	-	-
**5-Electrophysiological procedures (PM/ICD implantation-explantation)**	**1259**	**34**	**2.8**	**6**	**13**
**6-PAPVC repair**	**770**	**2**	**0.9**	**1.0**	**0.2**
PAPVC repair	731	2	1.0	1.0	0.2
PAPVC scimitar repair	39	-	-	-	-
**7-Fontan**	**333**	**13**	**9.8**	**29**	**14**

**8-Mechanical support implantation (ECMO, left/right assist device)**	**116**	**69**	**66**	**4**	**36**
**9-Transplant (heart/heart and lungs)**	**98**	**19**	**22**	**1.0**	**7.7**
**10-Palliative procedures (shunt, systemic to pulmonary)**	**42**	**10**	**32**	**3**	**27**
**11-Other procedures**	**447**	**20**	**7.6**	**24**	**13**

AoV: aortic valve; ASD: atrial septal defect; AVC: atrio-ventricular canal; CABG: coronary artery bypass graft; DCRV: double-chambered right ventricle; ECMO: extracorporeal membrane oxygenation; ICD: implantable cardioverter-defibrillator; LV: left ventricle; MV: mitral valve; PA: pulmonary artery; PAPVC: partial anomalous pulmonary venous connection; PM: pacemaker; PV: pulmonary valve; RV: right ventricle; RVOT: right ventricle outflow tract; TV: tricuspid valve; VSD: ventricular septal defect.
